# One year after gestational diabetes: metabolic changes and predictors of postpartum dysglycaemia

**DOI:** 10.1007/s00592-026-02657-w

**Published:** 2026-01-31

**Authors:** Alessia Gaglio, Yana Pigotskaya, Gabriele Rossi, Marco Mirani, Federico Giacchetti, Valeria Grancini, Valeria Maggi, Giovanna Mantovani, Irene Cetin, Emanuela Orsi, Veronica Resi

**Affiliations:** 1 Endocrinology Unit, Fondazione IRCCS Ca’ Granda Ospedale Maggiore Policlinico, Via Francesco Sforza 35, 20122 Milan, Italy; 2 Obstetric Unit, Fondazione IRCCS Ca’ Granda Ospedale Maggiore Policlinico, Milan, Italy; 3 Endocrinology, Diabetology and Andrology Unit, IRCCS Humanitas Research Hospital, Via Manzoni 56, Rozzano, 20089 Milan, Italy; 4 Department of Clinical Sciences and Community Health, University of Milan, Milan, Italy; 5 Diabetology and Nutritional Unit, Department of Specialist Medicine, ASST Santi Paolo e Carlo, Milan, Italy

**Keywords:** Gestational diabetes, Postpartum follow-up, Impaired glucose tolerance, Lifestyle, Breastfeeding

## Abstract

**Background:**

Women with previous gestational diabetes mellitus (GDM) are at high risk of developing type 2 diabetes mellitus (T2DM). Although early postpartum screening is recommended, metabolic changes occurring during the first year remain poorly characterized, and Italian guidelines do not include assessment at this time point.

**Aim:**

To evaluate glycaemic and metabolic changes one year after delivery in women with previous GDM and identify clinical and lifestyle predictors of postpartum glucose impairment.

**Methods:**

A cohort of 134 women with prior GDM was assessed at 6–12 weeks (T0) and one year postpartum (T1). Anthropometric, biochemical, nutritional, lifestyle, and quality-of-life parameters were collected. Dietary habits were evaluated using a 3-day food diary and the PREDIMED questionnaire; physical activity was assessed using the International Physical Activity Questionnaire (IPAQ). Logistic regression models were used to identify predictors of altered OGTT at T1.

**Results:**

At baseline, 32.9% of women showed altered OGTT; this increased to 38.8% at one year, while T2DM prevalence rose from 2.2 to 5.2%. Insulin therapy during pregnancy was the only independent predictor of dysglycaemia at T1 (OR 3.5, 95% CI 1.28–9.50, *p* = 0.015). Women with altered OGTT reported lower SF-36 scores in the domains “role limitations due to physical health” (*p* = 0.016) and “health change” (*p* = 0.030). Breastfeeding was associated with more favourable glucose outcomes (*p* = 0.009).

**Conclusions:**

One-year follow-up after GDM reveals early metabolic and psychosocial differences not detectable in the early postpartum period. Insulin therapy during pregnancy strongly predicts glucose impairment, highlighting the need for extended postpartum surveillance and targeted lifestyle interventions.

## Introduction

Gestational diabetes mellitus (GDM) is a common pregnancy complication and represents a major risk factor for later development of type 2 diabetes mellitus (T2DM) [1]. In 2021, approximately 21.1 million live births (16.7%) were affected by GDM worldwide, and the prevalence in Italy is estimated at 14.3% [2]. Although glucose intolerance typically resolves after delivery, women with previous GDM exhibit a markedly elevated risk of progression to T2DM, with reported conversion rates ranging from 2 to 12.5% within the first postpartum year [3–5]. Multiple cardiometabolic risk factors cluster in women with prior GDM, including overweight/obesity, hypertension, dyslipidaemia, and metabolic syndrome [6, 7]. A meta-analysis evaluating risk factors in women with GDM found that family history of diabetes increased risk by 70% and elevated postpartum BMI was associated with a 2-fold greater risk [8]. Independent of BMI, postpartum weight gain was identified as a risk factor [9]. Advanced maternal age was also associated with a 20% greater risk of T2DM. Moreover, other pregnancy-related factors were predictors of progression to T2DM. Insulin requirement was associated with a 3.7-fold greater risk and elevated HbA1c was associated with 2.6 greater T2DM risk [10, 11]. Moreover, maternal obesity at the onset of pregnancy has been proposed as a contributing factor that may increase the risk of developing depression or poor quality of life post-partum [12]. Lifestyle factors play a key role in modulating postpartum metabolic risk. Adherence to healthy dietary patterns—particularly the Mediterranean diet—has been associated with improved weight control, glycaemic regulation, and reduced incident diabetes. Likewise, regular physical activity supports insulin sensitivity and cardiometabolic health; however, adherence tends to decline after childbirth due to reduced time, childcare demands, and psychosocial barriers [13, 14].

Professional societies —including The American College of Obstetricians and Gynecologists ACOG [15], the American Diabetes Association (ADA) [16], The International Federation of Obstetrics and Gynaecology (FIGO) [17], and National Institute for Health and Care Excellence (NICE) [18]—recommend OGTT at 6–12 weeks postpartum and periodic lifelong screening every 1–3 years. Conversely, current Italian guidelines [19], focus solely on the early postpartum window and do not address assessment at one year. In Italy, adherence to postpartum follow-up after GDM is frequently inadequate, as highlighted by Dalfrà et al. [20], and further confirmed by the STRONG study [21], which identified high-risk subgroups and substantial gaps in postpartum screening, who reported significant dropout rates despite structured programs.

This study characterizes metabolic changes during the first postpartum year in women with a history of GDM and identifies clinical, behavioural, and psychosocial predictors of altered glucose tolerance. We performed comprehensive statistical analyses to investigate the interplay between anthropometric, lifestyle, and quality-of-life variables and their implications for clinical management.

## Materials and methods

### Study population

This single-center observational study included 134 women with a history of gestational diabetes mellitus (GDM) diagnosed according to the IADPSG criteria. These criteria utilize a 75-g oral glucose tolerance test (OGTT) with the following diagnostic thresholds: fasting plasma glucose ≤ 92 mg/dL, 1-hour glucose ≤ 180 mg/dL, and 2-hour glucose ≤ 153 mg/dL. Participants were evaluated at the Diabetes Unit of the Fondazione IRCCS Ca’ Granda Ospedale Maggiore Policlinico (Milan, Italy). Assessments were conducted at 6–12 weeks postpartum (T0) and at one year (T1). The study protocol was approved by the institutional Ethics Committee (ID 4613-4613_17.07.2024_P_bis). Written informed consent was obtained from all participants. Study procedures were performed in accordance with the Declaration of Helsinki ethical principles for medical research involving human subjects.

### Clinical and laboratory assessments

Anthropometric parameters (weight, height, BMI) [22], blood pressure, and biochemical variables (fasting glucose, HbA1c, lipid profile) were collected. OGTT was performed at both time points. Body composition was evaluated through bioelectrical impedance analysis (BIA), including measurements of total body water, fat-free mass, fat mass, and muscle mass.

### Lifestyle and dietary assessments

Dietary intake was assessed using a 3-day food diary and analysed using Metadieta^®^ Professional. Adherence to the Mediterranean diet was evaluated with the 14-item PREDIMED score [23]. Physical activity was measured using the International Physical Activity Questionnaire (IPAQ) [24]. Quality of life was assessed using the SF-36 questionnaire.

### Statistical analysis

Continuous variables are presented as mean ± standard deviation (SD), and categorical variables as absolute numbers and percentages. Between-group comparisons were performed using Student’s t-test or Mann–Whitney U test, as appropriate, while categorical variables were compared using chi-square or Fisher’s exact test. Univariate logistic regression analyses were conducted to explore associations between individual clinical, anthropometric, metabolic, lifestyle, and psychosocial variables and altered glucose tolerance at one year postpartum (defined as altered OGTT and/or type 2 diabetes). Variables entered into the multivariate logistic regression model were selected based on a combination of statistical and clinical criteria. Specifically, variables showing a significant or borderline association in univariate analyses (*p* < 0.10) and variables considered clinically relevant a priori according to existing literature (e.g. age, family history of diabetes, body composition parameters, and insulin therapy during pregnancy) were considered for inclusion. Given the limited number of dysglycaemic events, the number of covariates included in the multivariate model was deliberately restricted to minimise overfitting and ensure model stability. Odds ratios (ORs) and 95% confidence intervals (CIs) were calculated. A two-sided p-value ≤ 0.05 was considered statistically significant.

All analyses were performed using SPSS Statistics version 26.0 (IBM Corp., Armonk, NY, USA).

## Results

### Participant characteristics

**Fig. 1:**
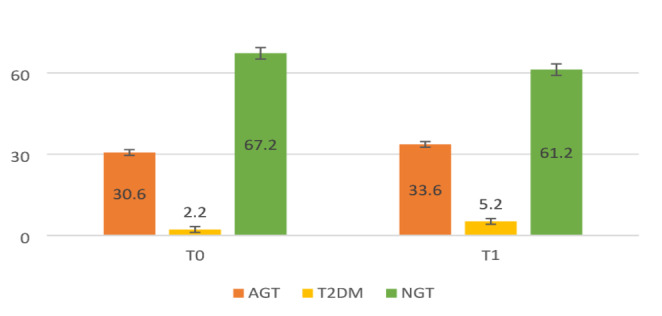
Distribution (%) of normal glucose tolerance (NGT), altered glucose tolerance (AGT), and type 2 diabetes (T2DM) at 6–12 weeks and one year postpartum

The cohort included 134 women with a mean age of 37.3 ± 4.6 years. Of these women, 76.5% were of Caucasian ethnicity. Pre-pregnancy BMI was 25.3 ± 4.7 kg/m² and current BMI 24.8 ± 4.9 kg/m² (Table 1). At baseline, 67.2% had normal glucose tolerance (90 women- group N) and 32.9% (44 women) presented altered OGTT (group A) (24 women with IFG, 14 with IGT, and 3 who exhibited both metabolic alterations). One year later this proportion increased to 38.8%, and T2DM prevalence rose from 2.2% to 5.2% (Fig. 1). BIA parameters did not differ significantly between women with normal (group N) and altered OGTT (group A). Body composition analysis by BIA was available for 89 participants. No significant differences were found in total body water (TBW), extracellular water (ECW), intracellular water (ICW), body cell mass (BCM), fat-free mass (FFM), fat mass (FM), or muscle mass (MM) between groups (all *p* > 0.05). 41% required insulin during pregnancy. In the univariate logistic regression model fat mass (FM) (OR = 1.06, *p* = 0.04) was associated with an increased risk of altered OGTT at one year. Adherence to the Mediterranean diet (PREDIMED score) remained moderate, while physical activity levels (IPAQ) tended to decrease (Table 2).

**Table 1:** Anthropometric and metabolic parameters at baseline (T₀) according to OGTT group

Variable		Normal OGTT (*N* = 90) mean ± SD		Altered OGTT (A = 44) mean ± SD		*p*-value
Age (years)		37.02 ± 4.43		37.57 ± 5.02		0.654
Pre-pregnancy weight (kg)		63.67 ± 14.14		63.43 ± 12.92		0.923
Pre-pregnancy BMI (kg/m²)		23.58 ± 5.09		28.93 ± 9.21		0.073
Weight at term (kg)		72.92 ± 13.51		71.93 ± 12.14		0.764
Weight gain in pregnancy (kg)		9.25 ± 13.86		8.50 ± 12.53		0.125
Weight at T0 (kg)		66.73 ± 13.68		64.89 ± 13.49		0.553
BMI at T0 (kg/m²)		24.66 ± 4.94		25.13 ± 4.88		0.406
Previous pregnancies (n)		0.78 ± 0.97		0.88 ± 1.07		n.s.
Systolic BP (CV, mmHg)		102.07 ± 10.90		100.37 ± 11.01		0.873
Diastolic BP (CF, mmHg)		86.77 ± 11.61		87.01 ± 13.14		0.412
HbA1c (%)		5.41 ± 0.32		5.38 ± 0.54		n.s.
Total cholesterol (mg/dL)		209.9 ± 40.8		198.9 ± 25.5		0.295
HDL (mg/dL)		67.0 ± 16.6		61.9 ± 16.9		0.192
Triglycerides (mg/dL)		94.5 ± 54.8		98.1 ± 52.0		0.775
LDL (mg/dL)		123.4 ± 37.0		117.8 ± 20.9		0.753
TSH (mIU/L)		1.75 ± 32.62		2.77 ± 1.92		0.878
TyG index		1306.8 ± 2144.4		2170.0 ± 2446.6		0.408

**Table 2:** Nutritional and lifestyle characteristics

Variable	Normal OGTT (*N*) mean ± SD	Altered OGTT (A) mean ± SD	*p*-value
Protein (g/kg)	1.13 ± 0.31	1.15 ± 0.37	0.919
Protein (%)	19.75 ± 4.01	20.32 ± 4.88	0.661
Lipids (%)	36.89 ± 7.17	35.61 ± 8.42	0.557
Carbohydrates (%)	43.54 ± 7.96	43.99 ± 10.34	0.925
Oligosaccharides/total CHO (%)	33.22 ± 10.59	32.09 ± 11.90	0.369
Kcal from oligosaccharides (%)	14.27 ± 4.76	13.60 ± 4.61	0.388
PREDIMED (score)	7.96 ± 1.87	7.91 ± 1.99	0.794
IPAQ (score)	704.6 ± 655.4	730.6 ± 596.5	0.713

### Determinants of glycaemic outcomes

Breastfeeding was significantly associated with normal glycaemic status (*p* = 0.009). Insulin therapy in pregnancy emerged as the strongest predictor of altered OGTT in both univariate and multivariate models (OR 3.5, 95% CI 1.28–9.50, *p* = 0.015). (Table 3).

**Table 3:** Logistic regression analysis: predictors of altered OGTT at 1 year

Variable	B	S.E.	Wald	*p*	OR (Exp B)	95% CI (lower–upper)
*Univariate analysis*						
Insulin therapy in pregnancy	1.273	0.397	10.27	**0.001**	3.57	1.64–7.78
IOM weight gain	–0.235	0.403	0.34	0.560	0.79	0.36–1.74
Family history of DM	0.726	0.389	3.48	0.062	2.07	0.96–4.43
Breastfeeding	–0.693	0.634	1.20	0.009	0.50	0.14–1.73
Hypertension	0.043	0.678	0.004	0.950	1.04	0.28–3.94
Age (years)	–0.014	0.040	0.13	0.724	0.99	0.91–1.07
Pre-pregnancy BMI (kg/m^2^)	–0.006	0.014	0.22	0.638	0.99	0.97–1.02
BMI at T0 (kg/m^2^)	0.030	0.039	0.61	0.436	1.03	0.96–1.11
CV (mmHg)	0.003	0.017	0.03	0.860	1.00	0.97–1.04
FFM (kg)	–0.036	0.023	2.47	0.116	0.96	0.92–1.01
FM (kg)	0.055	0.027	4.23	**0.040**	1.06	1.00–1.11
MM (kg)	–0.038	0.027	1.93	0.165	0.96	0.91–1.02
*Multivariate model*						
Insulin therapy in pregnancy	1.249	0.511	5.98	**0.015**	3.49	1.28–9.50
Fat mass (FM)	0.046	0.030	2.34	0.126	1.05	0.99–1.11
Family history of DM	0.610	0.504	1.47	0.226	1.84	0.69–4.95
Age (years)	0.003	0.050	0.003	0.960	1.00	0.91–1.11

### Quality of life

Women with altered OGTT reported significantly lower SF-36 scores in:

- Role limitations due to physical health (*p* = 0.016).
- Health change (*p* = 0.030).

No differences were identified in other domains (Table 4).

**Table 4:** Quality of life assessed by SF-36 questionnaire

Variable	Normal OGTT (*N*) mean ± SD	Altered OGTT (A) mean ± SD	*p*-value
SF-36: Physical functioning	97.18 ± 9.57	98.13 ± 6.45	0.513
SF-36: Role limitations	96.09 ± 18.08	80.65 ± 35.19	**0.016**
SF-36: Role limitations due to Emotional problems	73.66 ± 38.24	85.42 ± 29.26	0.171
SF-36: Energy/fatigue	48.47 ± 17.33	48.44 ± 16.09	0.832
SF-36: Emotional well-being	73.23 ± 16.89	72.75 ± 17.00	0.994
SF-36: Social functioning	85.08 ± 20.35	87.89 ± 17.24	0.481
SF-36: Pain	88.15 ± 19.94	93.20 ± 16.39	0.203
SF-36: Global Health	82.34 ± 13.38	76.05 ± 18.25	**0.030**

## Discussion

This study demonstrates that a substantial proportion of women with previous GDM experience glycaemic deterioration within the first postpartum year, even when early postpartum testing is normal. Nearly 40% of participants exhibited altered glucose tolerance at one year, and the prevalence of overt T2DM more than doubled. These findings corroborate prior evidence indicating that up to 10–20% of women with previous GDM develop glucose intolerance or diabetes within the first postpartum year [1, 25]. Consistent with prior evidence, insulin therapy during pregnancy was the strongest independent predictor of dysglycaemia (OR = 3.5, 95% CI 1.28–9.50, p = 0.015), reflecting underlying β-cell dysfunction. This observation reinforces the concept that insulin requirement during GDM reflects a more severe degree of beta-cell dysfunction, which persists beyond pregnancy. Identifying such women at the time of diagnosis may therefore help clinicians to tailor closer postpartum surveillance and lifestyle counseling. The observed increase of 16% in AGT and T2DM from 6–12 weeks to one year postpartum likely reflects persistent β-cell dysfunction unmasked by declining insulin sensitivity and lifestyle adherence over time, even in women who initially show normal glucose tolerance (NGT) [26]. Although body weight remained stable, physical activity tended to decrease and dietary quality did not improve, suggesting that behavioural and physiological factors jointly contributed to early metabolic deterioration. The predictive value of insulin therapy at T1 — also evident at T0 — supports its role as a marker of intrinsic β-cell impairment rather than a transient indicator of pregnancy severity. Although body composition and biochemical variables showed no significant inter-group differences, quality-of-life (QoL) assessments captured clinically meaningful disparities. Specifically, lower scores in ‘role limitations due to physical health’ and ‘health change’ suggest that subtle functional and psychosocial impairments may serve as early markers of metabolic vulnerability [27, 28]. Breastfeeding emerged as a protective factor, aligning with studies demonstrating improved insulin sensitivity and reduced long-term diabetes risk among lactating women [29, 30]. Yet, adherence to healthy diet and physical activity remained suboptimal, underscoring persistent barriers during the postpartum period. Similar behavioral patterns have been reported previously, with women describing barriers such as lack of time, motivation, and childcare responsibilities that limit engagement in lifestyle interventions [31, 32]. The persistence of sedentary behavior and suboptimal diet quality likely contributed to the observed metabolic deterioration. Our findings are consistent with real-world Italian evidence. The STRONG study [20] demonstrated that postpartum diabetes screening remains significantly suboptimal, with many high-risk women failing to complete OGTT testing. Similarly, Dalfrà et al. [21] reported relevant dropout rates even within structured follow-up programs. These converging data suggest that both clinical risk stratification and behavioural adherence barriers contribute to missed opportunities for early detection of dysglycaemia [33]. This study provides the opportunity to track long-term trends in metabolic alterations and to identify both the determinants of abnormal glucose tolerance (AGT) and the protective factors against the development of such impairments. These findings support the implementation of a structured follow-up at one year postpartum—currently absent from Italian recommendations—particularly for women treated with insulin. Integrating lifestyle counselling, psychosocial assessment, and breastfeeding support may help attenuate early metabolic deterioration. These women constitute a high-risk subgroup warranting targeted follow-up and early preventive intervention. Given the mean BMI of the cohort, the presence of alternative forms of diabetes, such as Type 1 Diabetes (T1D) or Maturity-Onset Diabetes of the Young (MODY), cannot be entirely excluded, although these conditions are considerably less prevalent. Future prospective studies and interventional trials, particularly those centered on lifestyle modifications, are essential to implement effective primary prevention strategies - specifically during the first postpartum year - to reduce incident T2DM and improve long-term cardiometabolic outcomes.

## Conclusions

One year after delivery, over one-third of women with previous GDM exhibited altered glucose tolerance. Insulin requirements during pregnancy emerged the most robust predictor of postpartum dysglycaemia, while breastfeeding and quality-of-life indicators were significantly associated with glycaemic outcomes. These findings underscore the necessity of comprehensive, long-term monitoring and lifestyle-based interventions that extend beyond the traditional early postpartum window.
